# Dietary Non-Drug Feed Additive as an Alternative for Antibiotic Growth Promoters for Broilers During a Necrotic Enteritis Challenge

**DOI:** 10.3390/microorganisms7080257

**Published:** 2019-08-13

**Authors:** Ali Calik, Islam I. Omara, Mallory B. White, Nicholas P. Evans, T. Peter Karnezos, Rami A. Dalloul

**Affiliations:** 1 Avian Immunobiology Laboratory, Department of Animal & Poultry Sciences, Virginia Tech, Blacksburg, VA 24061, USA; 2 Department of Animal Nutrition & Nutritional Diseases, Faculty of Veterinary Medicine, Ankara University, Ankara 06110, Turkey; 3 Department of Animal Production, Faculty of Agriculture, Cairo University, Giza 12613, Egypt; 4 PMI Nutritional Additives, Arden Hills, MN 55126, USA

**Keywords:** Broiler, microbiota, necrotic enteritis, prebiotic, probiotic

## Abstract

Necrotic enteritis, caused by *Clostridium perfringens,* is an enteric disease that leads to poor performance and increased mortality, resulting in significant economic losses in poultry production. This study evaluated the effects of a proprietary prebiotic, probiotic, and plant extract blend on performance of broilers during coccidiosis challenge leading to necrotic enteritis (NE). In total, 744 Cobb500 male broilers were randomly allocated to 3 treatments (8 replicates, 31 birds/pen) including, the negative control (NC) fed a basal diet; the positive control (PC) fed a basal diet with Virginiamycin; and the additive group fed basal diet with a blend of prebiotic, probiotic, and plant extract (BSN). A unique, naturally occurring NE model developed to mimic field conditions was implemented to challenge the birds. This model consists of spraying a concentrated commercial coccidiosis vaccine on litter and feed upon bird placement, which, in conjunction with the presence of *C*. *perfringens* spores in the environment, leads to the development of a NE outbreak one week post vaccine application. At the onset of NE on d7, three birds/pen were selected for scoring NE lesions. Body weight gain (BWG), feed intake (FI), and feed conversion ratio (FCR) were recorded on days 7, 14, 28, and 42. Carcass composition was assessed by dual energy X-ray absorptiometry (DXA) analysis on day 42. Dietary supplementation of BSN significantly (*p* < 0.05) improved FCR during starter and grower periods. Dietary treatments had no effect on NE lesions in the small intestine. DXA analysis revealed slightly higher lean content in BSN birds compared to NC. These results showed that dietary supplementation of the BSN blend significantly improved broilers performance during the early NE challenge phase, as well as in the grower period.

## 1. Introduction

Necrotic enteritis (NE) is a small intestinal inflammatory disorder in modern poultry production systems that causes poor performance, increased mortality, reduced welfare of the birds, and contamination of food products for human consumption [1,2]. In addition, necrotic enteritis has an extensive economic impact on the poultry industry, with an estimated annual total loss of more than $6 billion [3]. In poultry, both the clinical and subclinical causative agent of necrotic enteritis is *Clostridium perfringens* type A, which is normally found to be less than 10^2^ to 10^4^ CFU/g digesta in the small intestine of healthy birds [1,4,5]. At low population levels, *C*. *perfringens* is nonpathogenic [6], even high numbers are not sufficient to produce necrotic enteritis lesions in the small intestine [7]. However, specific dietary and environmental conditions, such as diets high in undigestible, water soluble polysaccharides or fish meal, co-infection with *Eimeria* spp., or immunosuppressive viruses can promote development of necrotic enteritis in chickens [8,9]. *Eimeria* infection is commonly used as a predisposing factor in experimental conditions which contribute to massive proliferation of *C*. *perfringens* [10]. *Eimeria*-assisted induction of necrotic enteritis may be related to the epithelial damage of the intestine, which causes release of serum and several nutrients [11] or increased mucin production [2], which provides a favorable growth conditions for *C*. *perfringens* colonization and proliferation.

Recent studies revealed that removal of antibiotic growth promoters (AGPs) and anticoccidial drugs in poultry feeding has led to a decrease in animal performance, an increase in feed conversion ratio (FCR), and incidences of necrotic enteritis [12,13]. Increasing concerns of consumers over the possible antibiotic residues in animal products and the emergence of antibiotic resistant microbes have highlighted the growing need for alternative feed additives such as probiotics, prebiotics, organic acids, plant extracts (e.g., essential oils), and others that modulate the gastrointestinal microbiota to improve performance and disease resistance [14]. Alternative feed additives exhibit their positive effects by increasing the numbers of beneficial bacterial in intestinal tract and promoting gut health [15]. Colonization of the intestine with beneficial microbes not only prevents pathogenic bacteria-related intestinal disorders, but also improves intestinal maturation and integrity [16].

Enteric diseases in commercial poultry production cause significant economic losses and threaten public health via the potential contamination of food products derived from these animals [17]. Thus, there is an urgent need to develop rational, alternative, and integrated management strategies not only to control, but to prevent necrotic enteritis [10]. Based on the previously reported favorable effects of alternative feed additives, such as probiotics, prebiotics, and phytogenics (alone or in combinations), the current study was designed to evaluate the effects of a combined feed additive (containing probiotic bacteria, prebiotic polysaccharides, plant extracts) on performance and response of broiler chickens to an early coccidiosis challenge as a predisposing factor to induce necrotic enteritis.

## 2. Results

### 2.1. Growth Performance

**Figure 1:**
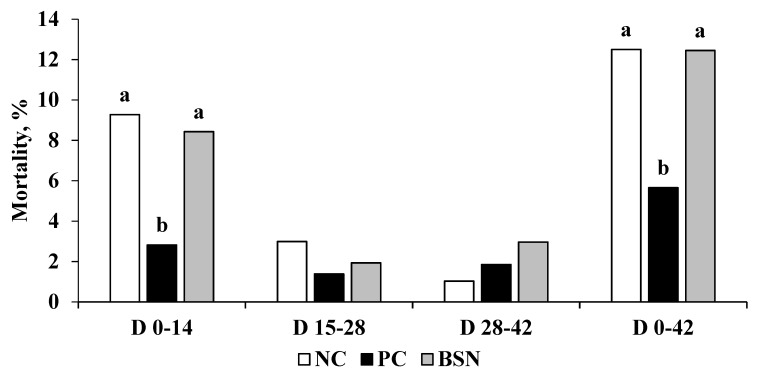
Effects of dietary non-drug feed additive (BSN) on mortality rate (%) of the birds during starter, grower, finisher, and overall experimental periods. Bars with different letters (a–b) differ significantly.

The effects of dietary supplementation of a non-drug feed additive on broiler growth performance are presented in Table 1 (BWG, g/bird; FI, g/bird; FCR, g/g bird) and Figure 1 (Mortality, %). No significant differences were observed in BWG during the starter (0–14 day) or overall trial period (0–42 day). However, birds in the BSN group tended to have higher (*p* = 0.051) BWG during the grower period (15–28 day). Antibiotic treatment (PC) increased the feed intake during the starter period as compared with NC and BSN groups (*p* = 0.005). Except for the starter period, no significant differences were observed between the groups in terms of FI during the entire study. The FCR was improved in birds fed the diet supplemented with a feed additive (BSN group) during the first week (*p* = 0.005), grower (*p* = 0.004), and finisher period *(p* = 0.048). However, no significant difference in FCR was observed over the cumulative study period. Only the dietary antibiotic treatment significantly reduced the mortality rate during the starter (*p* = 0.002) and overall experimental *(p* = 0.006) periods.

### 2.2. Lesion Scores

**Figure 2:**
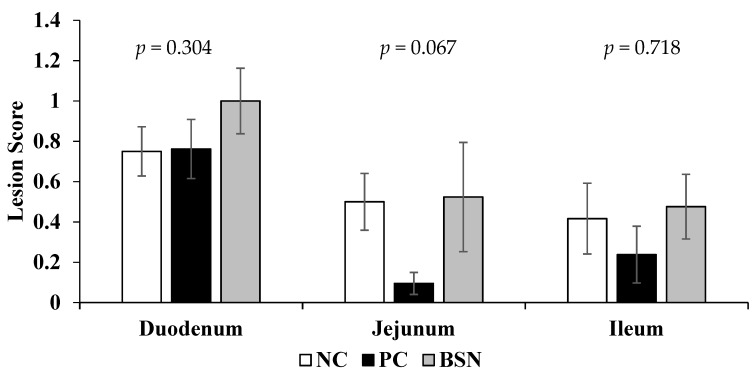
Effects of dietary non-drug feed additive (BSN) on lesion scores of duodenum, jejunum, and ileum on day 7. Each bar represents the mean ± SE values of 24 birds per treatment.

The effect of dietary supplementation of the non-drug feed additive on intestinal lesion scores is presented in Figure 2. No significant differences were observed in duodenal and ileal lesion scores between the treatments. However, jejunal lesion scores were minimal for all groups and tended to be lower in PC birds when compared with NC and BSN birds on day 7 (*p* = 0.067).

### 2.3. Carcass and Body Composition

**Figure 3:**
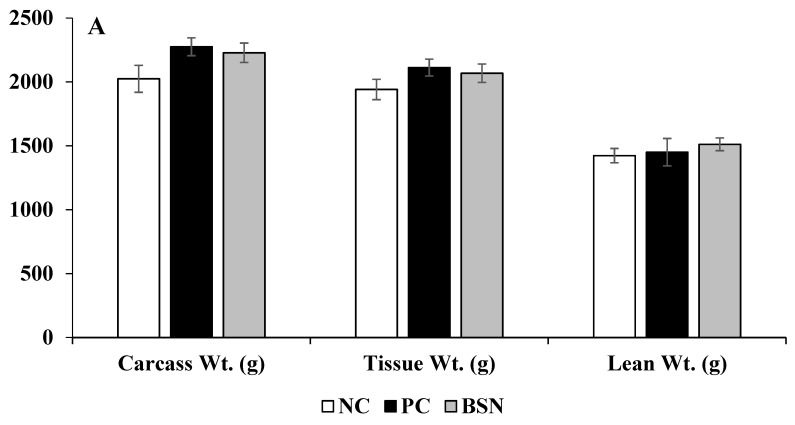

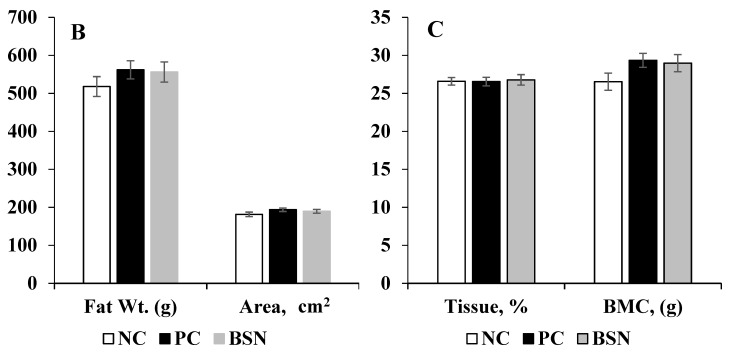
Effects of dietary non-drug feed additive (BSN) on body composition of broilers. (**A**) Carcass weight (g), tissue weight (g), and lean weight (g). (**B**) Fat weight (g) and body surface area (cm^2^). (**C**) Tissue (%) and bone mineral content, BMC (g).

The effects of dietary supplementation of non-drug feed additives on broiler body/carcass compositions (carcass weight, tissue weight, lean weight, fat weight, surface area of defeathered, whole carcass, tissue percentage, and bone mineral content) are shown in Figure 3A–C. No significant differences were observed between the treatment groups in terms of body/carcass composition on day 42 (*p* > 0.05).

## 3. Discussion

Among the strategies to reduce the use of antibiotics in the broiler industry, dietary supplementation of non-drug feed additives is becoming an accepted alternative because of their beneficial effects against intestinal pathogens and improved bird performance and health without having the risk of drug residues or antibacterial resistance. In this context, the present study investigated the effect of a novel non-drug feed additive blend on broiler performance, body composition, and intestinal lesion scores of broiler chickens during a coccidiosis challenge leading to necrotic enteritis similar to field conditions. The more commonly published NE models involve first experimentally inoculating birds orally with a high dose of live *Eimeria* oocysts, followed with consecutive doses of *C*. *perfringens* bacteria. Conversely, the unique model presented herein relies on the bacterial spores present in the barn environment in addition to commensal *C*. *perfringens* under certain circumstances.

As an important intestinal infectious disease, necrotic enteritis is responsible for reduced performance and increased mortality in birds [18]. The presented results showed that dietary supplementation of a feed additive blend did not alleviate the growth suppression effect of NE during the starter period (days 0–14). However, birds fed a diet supplemented with BSN and AGP (Virginiamycin) had significantly better FCR during days 15–28 and days 15–42, which show that this feed additive promoted broiler performance similar to the AGP. However, dietary supplementation of BSN was significantly less effective than antibiotic treatment in reducing the mortality rate during the starter period (days 0–14) when the challenge was implemented. On the other hand, no significant mortality was observed among the treatment groups during days 15–28 and days 15–42. Dietary supplementations of certain feed additives, such as probiotics [19,20], prebiotics [21], essential oils [22], or mixed combination products [23], can be effective at reducing the negative effects of necrotic enteritis by modulating the beneficial intestinal microbiota and improving intestinal integrity and immune status of broiler chickens [18]. However, young chickens are more susceptible to intestinal infections since the protective microbial colonization patterns are unstable in early life stages [24]. Hence, the reason behind the reduced performance and increased mortality in BSN birds compared to the AGP during the starter period was likely due to the less-established microbiota early on during the first two weeks of the study. Moreover, even though we observed better FCR in BSN and AGP birds during the grower and finisher periods, numerically higher FCR in the AGP treatment and increased mortality in the BSN group during the starter period might be the reason for the non-significantly different overall corrected FCR (days 0–42).

Broiler performance and intestinal lesion scores are important parameters used to evaluate the severity of enteric diseases such as coccidiosis [25] and NE [26]. Our results showed that dietary treatments had no effect on lesion scores in the duodenum and ileum. Conversely, jejunal lesion scores tended to be lower (*p* = 0.067) in PC birds compared to NC and BSN birds; albeit all three groups exhibited relatively minimal lesions. This outcome coincides with the observed higher feed intake and lower mortality in PC birds. Less intestinal damage is directly related to improved nutrient absorption and a better intestinal barrier against pathogens [25]. These results partially concur with the findings of M’Sadeq, Wu, Choct, Forder and Swick [15], who reported that antibiotic-treated birds had lower duodenal lesion scores than non-medicated and prebiotic-treated birds. Contradictory results are also evident in previous reports with regard to a decrease in intestinal NE lesion scores in broilers fed diets with feed additives [26,27]. The methodology employed in this study is quite unique, whereby the model does not involve inoculating *C*. *perfringens* bacteria, hence it is more comparable to field conditions. Conversely, in the reported studies, birds were directly inoculated towards the end of the starter period and lesion scores were evaluated during the grower period. These differences might be the underlying reason for the slightly higher lesion scores in the BSN group.

Dual-energy X-ray absorptiometry is used to estimate fat, fat-free soft tissue, and bone mineral components of the body [28]. For broiler studies, it is a useful tool for the in-depth understanding of bird performance [29]. Carcass, tissue, and lean weights showed similar patterns to overall BWG (days 0–42). However, there is limited reporting on the effects of such feed additives on broiler body composition during a pathogenic challenge. Although not significantly different, PC and BSN treatments had higher lean body composition than NC treatment, which is important from an industry perspective.

In conclusion, the present study demonstrated that supplementation of a feed additive blend significantly improved FCR during the grower and finisher periods when birds were subjected to a naturally occurring necrotic enteritis challenge. However, no significant differences were observed in overall performance, carcass composition, and lesion scores as compared with the control group. It should be noted that the severity of the infection or the supplementation level are important when alternative growth stimulators are applied. It would be useful to further assess the effects of this product on specific intestinal immune responses, tight junction proteins, intestinal microbiota, and short chain fatty acid composition under similar disease challenge conditions.

## 4. Materials and Methods

### 4.1. Birds, Diet, and Management

This project was approved and conducted under the guidelines of the Virginia Tech Institutional Animal Care and Use Committee (IACUC #16-107; Approved July 8 2016). On day of hatch, 744 Cobb500 male broiler chicks were randomly allocated to 3 experimental dietary groups (8 replicate floor pens, 31 birds/pen) as follows: The negative control (NC) fed a corn/soybean basal diet; the positive control (PC) fed a basal diet with Virginiamycin; and the additive group (BSN) fed the basal diet supplemented with a feed additive (567 g/ton for starter, 454 g/ton for grower, and 340 g/ton for finisher) consisting of essential oils (*Yucca schidigera* extract), prebiotic polysaccharides from food grade yeast extracts, glucans, mannans, quitines, and galactans, plus saponins obtained from the *Yucca schidigera* plant and a combination of probiotic *Bacillus* bacteria (*B*. *subtilis*, *B*. *licheniformis*, *B*. *coagulans*), delivering 1.32 × 10^11^ CFU/kg of total microbial count. Chicks had ad libitum access to water and a non-medicated corn/soy-based crumble (starter days 1–14) or pellet form diets (grower days 15–28 and finisher days 29–42). The corn/soybean basal diet (Table 2) was formulated to meet or exceed the nutrient requirements for broilers as recommended by the National Research Council (NRC) [30]. The temperature was maintained at 34 °C from day 1 to day 7 post-hatch and was progressively reduced to 27 °C on day 14, 21 °C on day 28, and 18 °C on day 42. The light cycle was 20 h light and 4 h dark.

### 4.2. Necrotic Enteritis Challenge and Lesion Scoring

Upon placement, all birds were indirectly challenged with a coccidia vaccine as a predisposing factor to NE. This is a unique, naturally occurring NE model developed on our research farm which consists of spraying a concentrated commercial coccidiosis vaccine on litter and feed upon bird placement, which, in conjunction with the presence of *C*. *perfringens* spores in the barn environment, leads to the development of a NE outbreak one week post vaccine application. For this trial, we applied the Coccivac^®^-B52 vaccine (containing live oocysts of *Eimeria acervulina*, *E*. *maxima*, *E*. *maxima* MFP, *E*. *mivati*, and *E*. *tenella*; Merck Animal Health). Three birds per pen were selected (based on average pen weight) on day 7 (based on NE peak outbreak), individually weighed, and sacrificed. Intestinal NE lesion scores were assessed for the afflicted sections of the small intestine (duodenum, jejunum, and ileum), per standard protocols by personnel blinded to the treatments using the following scale: A value of 0 = no gross lesions, 1 = thin-walled or friable, 2 = focal necrosis or ulceration, 3 = multifocal coalescing areas (large patches) of necrosis, and 4 = severe extensive necrosis.

### 4.3. Growth Performance

Standard performance parameters including body weight (BW), body weight gain (BWG), and feed intake (FI) were recorded on days 7, 14, 28, and 42 on a bird per pen basis. Daily mortality and body weight of each dead bird were recorded and feed conversion ratio (FCR) was corrected by accounting for the BW of each dead bird.

### 4.4. Carcass and Body Composition

On day 42, two birds per pen were individually wing-banded, sacrificed by cervical dislocation, and subsequently defeathered and stored at −20 °C until further analysis. Carcasses were thawed and scanned by dual energy X-ray absorptiometry (DXA) using the GE Healthcare Lunar Prodigy Advance, System ID PA+130,744 (General Electric, Madison, WI, USA). After scanning, the Prodigy Small Animal Software was used to quantify and calculate whole, defeathered carcass weight (g), lean weight (g), fat weight (g), tissue weight (lean weight, g + fat weight, g), and carcass surface area (cm^2^), tissue (%), and bone mineral content, BMC (g).

### 4.5. Statistical Analysis

Performance and carcass composition data were subjected to a one-way analysis of variance (ANOVA) using the SAS program (SAS, 2004). When significant differences were noted, Tukey’s test was used to compare separated means. The influence of the feed additive on NE lesion scores was performed with the non-parametric Kruskal-Wallis test. Mortality rates were compared using a Chi-square test. Statistical differences were considered significant at *p* ≤ 0.05.

## Figures and Tables

**Table 1:** Effects of dietary non-drug feed additive (BSN) on body weight gain (g/bird), feed intake (g/bird), and feed conversion ratio (g/g bird) of the experimental birds.

	Dietary Treatments			Statistics	
	NC	PC	BSN	SEM	*p*-Value
**0 to 7 d**					
BWG	111.74	112.46	114.22	1.56	0.815
FI	145.57	151.21	139.14	2.10	0.056
FCR	1.303^ab^	1.350^a^	1.223^b^	0.02	0.005
**0 to 14 d**					
BWG	311.27	325.79	314.44	5.92	0.596
FI	473.58^b^	523.09^a^	462.40^b^	8.64	0.004
FCR	1.528	1.619	1.475	0.03	0.082
**15 to 28 d**					
BWG	771.53	874.56	906.39	24.17	0.051
FI	1451.3	1495.0	1483.6	27.94	0.817
FCR	1.906^a^	1.711^b^	1.643^b^	0.04	0.004
**15 to 42 d**					
BWG	1692.0	1902.7	1856.9	47.95	0.171
FI	3360.9	3603.1	3506.1	72.46	0.407
FCR	2.00^a^	1.894^b^	1.894^b^	0.02	0.048
**0 to 42 d**					
BWG	2003.3	2228.5	2171.3	52.16	0.191
FI	3834.4	4126.2	3968.6	77.15	0.316
FCR	1.925	1.854	1.833	0.02	0.102

**Table 2:** Composition and calculated analyses of basal diet.

Ingredient, %	Starter (0–14 d)	Grower (15–28 d)	Finisher (29–42 d)
Corn	60.14	65.34	69.91
Soybean meal (48%)	22.41	16.43	10.76
Distiller’s grain	7.00	8.00	9.00
Poultry by-product meal	5.00	5.00	4.00
Grease (yellow)	1.90	2.12	2.76
Limestone	0.58	0.54	0.70
Dicalcium phosphate	1.15	0.90	0.78
Salt	0.27	0.17	0.16
DL-Methionine	0.18	0.30	0.80
l-Lysine	0.63	0.60	0.80
l-Threonine	0.10	0.10	0.10
Vit. & Min. Premix (Big Spring Mills) ^1^	0.63	0.50	0.50
Total	100.00	100.00	100.00
**Calculated nutrient level**			
ME, kcal/kg	3,036	3,102	3,157
CP, %	21.00	19.00	17.00
Ca, %	0.90	0.80	0.76
Available P, %	0.45	0.40	0.35
Total P, %	0.71	0.64	0.57
Lysine, %	1.50	1.33	1.32
Methionine, %	0.50	0.60	1.06
Threonin, %	0.89	0.81	0.71
Tryptophan, %	0.22	0.19	0.16

^1^ Provided per kilogram of diet: Cobalt (min), 30 ppm; copper (min), 4.75 ppm; iodine (min), 1.18 ppm; iron (min), 59.40 ppm; manganese (min), 75.50 ppm; zinc (min), 57.20 ppm; vitamin A (min), 7749.28 IU; vitamin D_3_ (min), 2596.01I CU; vitamin E (min), 1.94 IU; vitamin B_12_ (min), 0.01 mg; menadione (min), 1.36 mg; riboflavin (min), 4.84 mg; D-pantothenic acid (min), 7.13 mg; niacin (min), 23.25 mg; choline (min), 448.43 mg.
